# “Get Me Better!”–Catatonia, or FND: A Diagnostic Case Study

**DOI:** 10.1192/bjo.2026.11771

**Published:** 2026-06-30

**Authors:** Obumneme Chinweuba, Aaliya Majeed

**Affiliations:** Kent and Medway NHS Mental Health Trust, Maidstone, United Kingdom

## Abstract

**Aims:**

Catatonia is a complex neuropsychiatric syndrome with various neuropsychiatric manifestations. It has long been known as a feature of schizophrenia and bipolar disorders. Catatonia has now been recognized as a trans-diagnostic neuropsychiatric syndrome with a wide range of psychiatric and medical aetiologies.

Functional Neurological Disorder (FND) presents with neurological symptoms not consistent with known neurological disorders and often with a background of psychological stressors. The overlap between catatonia and FND with regards to presenting features such as mutism, motor disorders and fluctuating course, can cause a diagnostic challenge. Catatonia and FND can coexist, adding further complexity.

We present a case of recurring catatonia in an elderly individual which highlights diagnostic challenges and the importance of long-term assessment.

**Methods:**

A 73-year-old woman with no previous psychiatric history was seen on three separate occasions over a seven-month period with recurring episodes of neuropsychiatric symptoms. Her medical history includes hypertension, COPD, and kyphosis.

First presentation:

She was admitted to the Emergency Department (ED) with acute onset of mutism, food refusal, confusion, wandering, and paranoid delusions. She was admitted to a stroke unit under neurology due to suspected stroke. Extensive investigations over a 21-day period, including CT and MRI scans, revealed small vessel diseases and age-appropriate global involutional changes. Liaison Psychiatry Service review suggested a diagnosis of delirium. Her symptoms resolved spontaneously, and she was discharged home.

Second presentation (four months later):

She presented to the ED with a four-day history of sudden mutism, urinary incontinence, and significant decline in self-care. She was admitted and treated for a urinary tract infection. Extensive workup failed to show any abnormality. Her symptoms resolved spontaneously during admission. The diagnosis of Functional Neurological Disorder was made, and she was referred to the Neuropsychiatry service. The referral was declined due to diagnostic uncertainty.

Third presentation (two months later):

She presented with sudden onset mutism and limb weakness. Her husband gave a history of similar episodes occurring intermittently over the past three years. These episodes were characterized by sudden onset, a three-week duration, and a possible temporal relation to psychosocial stressors. Previously, she had been able to manage these episodes at home with her husband. Increasing frequency and severity of episodes prompted hospital presentation.

Investigations were again negative. LPS assessment revealed mutism and significant negativism. The lorazepam challenge test revealed a partial yet clear response. This prompted a revision of diagnosis from FND to catatonia.

She was started on lorazepam and sectioned under Section 2 of the Mental Health Act. Her catatonic symptoms resolved with improvement in negativism. Her mutism progressed from global mutism to selective mutism. Subsequently, affective and psychotic symptoms were noted. These included low mood, delusional misidentification in the form of hospital staff being replaced by actors and that she was in danger.

She was commenced on sertraline and quetiapine, which was later changed to aripiprazole. She was transferred to a mental health unit. She made good adjustment to psychological and occupational therapy interventions. She was discharged after about four weeks and has remained well with community mental health team’s support.

**Results:**

Despite being well characterized clinically; the pathophysiology of catatonia is not well understood. Functional neuroimaging studies have implicated dysfunction in the cortico-striato-thalamic and the cortico-cerebellar system. The hyperactive prefrontal cortex has been implicated in psychiatric catatonia, with hyperactive motor cortices also implicated. Conversely, hypoactive regions have been implicated in medically related catatonia. These studies emphasize the heterogeneity of catatonia and the limitation of the current diagnostic system, which views it as a feature of schizophrenia spectrum disorders.

The increasing recognition of catatonia in neurodevelopmental disorders, affective disorders, and medical conditions makes diagnosis difficult. It can be mistaken for FND, particularly if symptoms are cyclical, related to stress, and not associated with abnormal investigations. This case emphasizes the importance of a long history, collateral information gathering, and benzodiazepine challenge test in diagnosis.

Benzodiazepines, particularly lorazepam, remains first-line drug, with electroconvulsive therapy a choice for refractory cases. There is emerging evidence for the use of NMDA receptor antagonists such as memantine.

**Conclusion:**

Catatonia can mimic FND, making diagnosis difficult. This case highlights the importance of maintaining a high index of suspicion, especially if symptoms presents in a cyclical manner with spontaneous resolution. The Bush-Francis Catatonia Rating Scale is an important tool in diagnosis. Diagnosis requires close cooperation between clinicians from psychiatry, neurology, and psychology.

